# Effect of Temperature-Induced Aging on the Gas Permeation Behavior of Thin Film Composite Membranes of PIM-1 and Carboxylated PIM-1

**DOI:** 10.1021/acs.iecr.4c02230

**Published:** 2024-09-04

**Authors:** Ming Yu, Andrew B. Foster, Mustafa Alshurafa, Colin A. Scholes, Sandra E. Kentish, Peter M. Budd

**Affiliations:** † Department of Chemical Engineering, The University of Melbourne, Melbourne, VIC 3010, Australia; ‡ Department of Chemistry, School of Natural Sciences, The University of Manchester, M13 9PL Manchester, U.K.

## Abstract

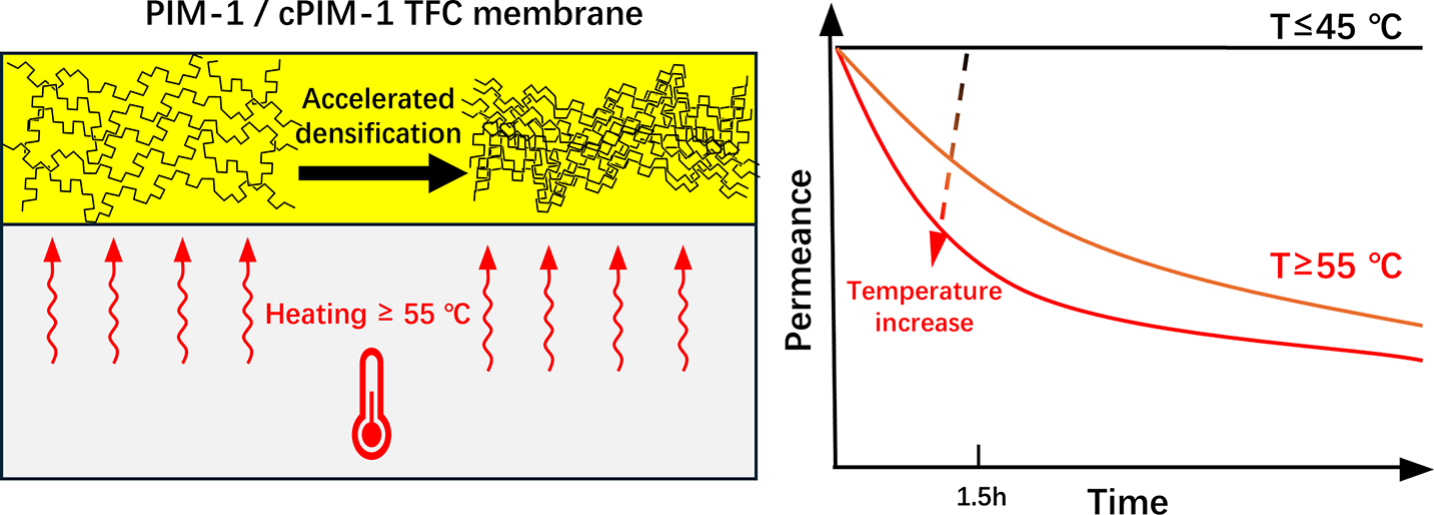

Polymers of intrinsic microporosity (PIMs) are a class of promising gas separation materials due to their high membrane permeabilities and reasonable selectivities. When processed into thin film composite (TFC) membranes, their high gas throughput aligns closely with industrial requirements, but they are prone to physical aging and plasticization effects. TFC membranes based on the prototypical PIM-1 and its carboxylated derivative cPIM-1 exhibit temperature-dependent gas permeation behavior, which has not been extensively studied before. In single CO_2_ permeation tests, measurable physical aging occurred when the temperature was raised to 55 °C within a period of 90 min, and the aging rate accelerated as temperature was raised further. TFC membranes prepared from cPIM-1 exhibited a faster aging rate compared to PIM-1 at the same temperature. The decreased permeance could be at least partially recovered through a 5 day methanol vapor treatment. In mixed gas experiments, all membranes showed decreased permselectivities at elevated temperatures. The plasticization pressure of TFC membranes occurred at around 1 bar of CO_2_ partial pressure, independent of temperature. Significant plasticization was particularly evident for cPIM-1 TFC membranes under CO_2_/CH_4_ conditions with increasing temperature, which resulted in increased gas permeance for both components.

## 1 Introduction

Polymers of intrinsic microporosity (PIMs) make up a class of highly glassy polymers, which were first reported by Budd and McKeown in 2004.^1^ The highly rigid and contorted polymer backbone endows PIMs with very high free volume, and thus high gas permeabilities and reasonable gas pair selectivities, making PIMs ideal membrane materials for postcombustion carbon capture^2^ and natural gas sweetening.^3^ In industrial applications, membranes with minimum gas transport resistance and maximized gas throughput are required, such as thin film composite (TFC) membranes,^4,5^ which consist of a thin active layer, with a thickness below 2 μm, coated onto a porous substrate, providing necessary mechanical support with minimum resistance.

The prototypical PIM, termed PIM-1, is a thermally stable polymer with a high glass transition temperature^6^ and no obvious degradative weight loss below 450 °C.^7,8^ However, as evidenced by Song et al.^9^ and Tian et al.,^10^ thermal treatment around 350–400 °C is sufficient to induce cross-linking of PIM-1 membranes, resulting in densified polymeric structures and improved molecular sieving properties. The dielectric behavior of a PIM-1 membrane was found to be reversible in the temperature range from −100 to 200 °C.^11^ However, when a PIM-1 sample was further heated to 250 °C, an irreversible decreased dielectric loss was observed in the following cooling/heating cycles, accompanied by a slight 1.2 wt % weight loss, implying a thermally induced structural change.^12^ Furthermore, PIM-1 membranes also exhibit temperature-dependent behavior in terms of positron lifetime, reaching a maximum value around 90–110 °C, ascribed to a thermally activated and reversible contraction of the ladder polymer backbone around the spirocenters.^13^ The thermal expansion coefficient was found to be almost constant below 110 °C.^14^ In recent work reported by Yamato et al.,^15^ PIM-1 self-standing membranes (30–70 μm) exhibited accelerated polymer relaxation at temperatures above 95 °C.

Thermal annealing of PIM-1 thin films (<1 μm) resulted in a reduction in film thickness, but a 2 h annealing treatment at 150 °C seemed to have no effect on heptane permeance in nanofiltration tests,^8^ indicating a structural change that can be recovered by the swelling in heptane. Bernardo et al.^16^ reported that PIM-1 self-standing membranes exhibited different physical aging behaviors after thermally treating them at different temperatures (25, 75, and 125 °C) for 4 h at the start. The changes in gas permeation performance induced by physical aging can be mitigated through thermal treatment, as the structural changes and reduced permeabilities at temperatures <125 °C can be correlated to an accelerated physical aging effect, since membranes under both normal physical aging and temperature treatment showed similar changes in X-ray scattering (XRD) patterns.^17^ Lee et al.^18^ prepared a hyperaged Trip (Me_2_)-TB TFC membrane by subjecting it to a 90 °C vacuum treatment for 4 h, which exhibited a similar XRD spectrum and gas separation performance to films that were normally aged for 336 h.

Li et al.^19^ comprehensively studied the temperature-dependent gas separation behavior of self-standing PIM-1 membranes under single gas conditions within the temperature range from 25 to 55 °C. The change in temperature affected both the diffusivity and solubility parameters. For less condensable gases (N_2_, O_2_, H_2_, He) the diffusivity term dominated the permeability, but for more sorbing gases (CO_2_ and light hydrocarbons), the permeability depended more on the solubility parameter. Interestingly, PIM-1 membranes exhibited surprisingly high permeability selectivities at subambient temperatures,^20^ benefiting from significantly enhanced diffusivity selectivity but relatively unchanged solubility selectivity. A significant reduction in aging rate was observed for PIM-1 self-standing membranes when stored at −20 °C.^21^ In mixed gas tests, gas separation performance was generally less good due to the competitive sorption effect, typically resulting in lower gas permeabilities and selectivities.^22^ Furthermore, the performance of the TFC membranes might be even worse. Physical aging typically leads to a reduction of membrane permeability caused by structural densification, while plasticization results in an increase in membrane permeability but drop in selectivity due to membrane swelling induced by the highly soluble penetrating components,^23^ and thinner active layers are more vulnerable to both physical aging^24^ and plasticization^25^ compared to self-standing membranes.

Recently, chemical postmodifications on PIM-1, such as amination,^26,27^ sulfonation,^28^ amidoximation, and carboxylation,^29^ have drawn attention as they usually lead to a better gas separation performance than the parent PIM-1. However, only a limited number of modified PIM-1s can be successfully fabricated into TFC membranes, as this requires the polymer material to have a good solution processability. We have prepared carboxylated PIM-1 (cPIM-1) TFC membranes which exhibited similar CO_2_ permeance to PIM-1, but much higher selectivities under single gas conditions.^7^ However, significant plasticization of cPIM-1 decreased the mixed gas performance. Nevertheless, cPIM-1 prepared by acid hydrolysis is still considered a promising material and is included in other works, such as surface-engineered PIM-1 membranes,^30^ preparation of high-performance MOF fillers^31,32^ toward CO_2_ capture, investigation of natural gas sweetening in a MMM system,^33^ and as precursors for preparing cross-linked TFC membranes in nanofiltration.^34,35^ In postcombustion carbon capture processes, the temperature of flue gas after cooling might still be within the range from 40 to 75 °C,^36^ so it is important to study the gas separation behavior of membranes at above-ambient temperatures.

**Figure 1:**
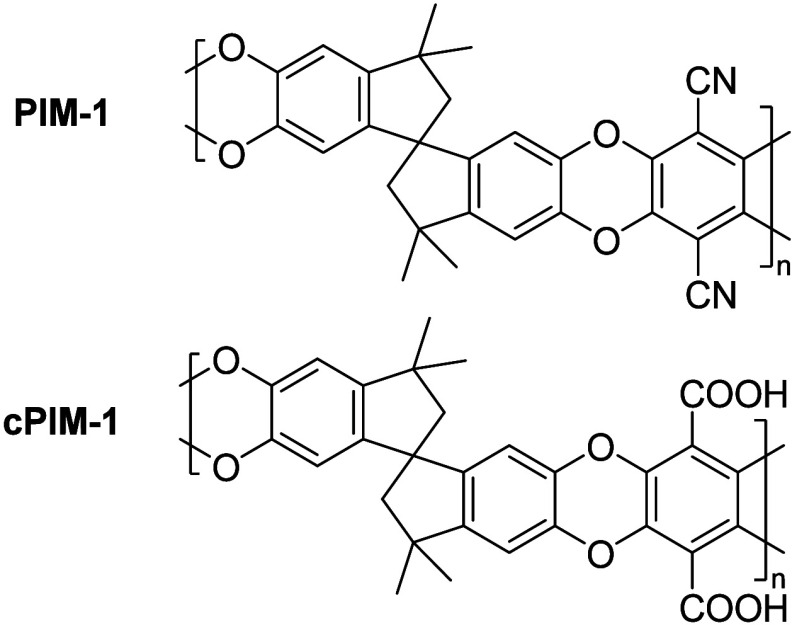
Chemical structures of PIM-1 and cPIM-1.

In the present work, we prepared TFC membranes of PIM-1 and cPIM-1 (structures shown in Figure 1) via a kiss-coating process to study the temperature dependence of gas separation behavior within the range from 25 to 85 °C under both single gas and mixed gas conditions. TFC membranes exhibited accelerated physical aging at elevated temperatures. The aging rate increased as temperature increased further. The mixed gas separation performance was typically reduced at elevated temperatures, exhibiting significantly decreased permselectivities. CO_2_ permeation experiments at low partial pressure helped to determine the critical plasticization pressure, which was independent of the temperature and applied to all polymers used in this work.

## 2 Experimental Section

### 2.1 Materials

5,5′,6,6′-Tetrahydroxy-3,3,3′,3′-tetramethyl-1,1′-spirobisindane monomer (TTSBI, 97%) was purchased from Alfa Aesar and purified further before use as described below. Tetrafluoroterephthalonitrile monomer (TFTPN, >99%) was purchased from Fluorochem and used as received. Potassium carbonate (K_2_CO_3_, anhydrous, ≥99.5%), tetrahydrofuran (THF, analytical reagent grade, ≥99.8%), sulfuric acid (H_2_SO_4_, laboratory reagent grade, ≥95%), and glacial acetic acid (analytical reagent grade, ≥99.7%) were purchased from Fisher Scientific. Chloroform (HPLC, ≥99.8%), ethyl acetate (GC, ≥99.5%), hexane (HPLC, ≥97%), toluene (ACS reagent, ≥99.7%), *N*,*N*-dimethylacetamide (DMAc, anhydrous, 99.8%), acetone (for analysis), methanol (ACS reagent, ≥99.8%), 1,4-dioxane (anhydrous, 99.8%), chloroform-*d* (99.8 atom % D), and glass wool were purchased from Sigma-Aldrich. Dimethyl sulfoxide-*d*_6_ (DMSO-*d*_6_, D, 99.9%) was purchased from Cambridge Isotope Laboratories, Inc. Polyacrylonitrile (PAN) ultrafiltration membrane support (UF010104 batch G) was purchased from SolSep BV (The Netherlands).

### 2.2 Polymer Synthesis and Purification

The TTSBI monomer was purified before use.^7^ 20 g of TTSBI was added into 333 mL of ethyl acetate and refluxed at 90 °C for 2 h with the system purged with N_2_, and then another 333 mL of hexane was added. After 10 min, the system was cooled in ice for 3 h. Purified TTSBI was collected by vacuum filtration and dried under vacuum for another day. TFTPN monomer was vacuum-dried 1 day before use.

PIM-1 was synthesized based on a high-temperature synthesis method.^37^ 17.03 g (50 mmol) of TTSBI, 10 g (50 mmol) of TFTPN, and 20.73 g (150 mmol) of K_2_CO_3_ were added to a 500 mL three-neck round-bottom flask and purged with N_2_. After 180 mL of a solvent mixture (120 mL of DMAc and 60 mL of toluene) was added, the flask was placed on a hot plate (IKA, UK), which was turned on immediately with a set point of 160 °C. Stirring was provided by an overhead stirrer (Heidolph Instruments Hei-TORQUE Expert 100, Germany) with the stir speed gradually increased to ensure a uniform mixing. Two batches of additional solvent mixture (30 mL) were added during the reaction. After 30 min, the reaction was quenched with an excess amount of methanol. Raw PIM-1 polymer was filter collected and redissolved in 700 mL of chloroform and reprecipitated in methanol again. The polymer was then refluxed in deionized water overnight to remove salts. Later, the polymer was immersed in dioxane, washed with acetone and methanol, and then immersed in methanol overnight. Finally, PIM-1 was dried at 120 °C under a vacuum for 2 days.

An acid hydrolysis of PIM-1 was conducted based on previous work.^29^ 4.8 g of PIM-1, 288 mL of H_2_SO_4_, 96 mL of glacial acetic acid, and 288 mL of deionized water was added into a round-bottom flask. The system was heated at 150 °C for 12 or 24 h. Then the solution was cooled and neutralized in 8 L of deionized water. The product (cPIM-1) was then collected and refluxed in a slightly acidic environment overnight. Finally, cPIM-1 was collected and dried at 120 °C under vacuum for 2 days.

### 2.3 Polymer Characterization

Proton nuclear magnetic resonance (^1^H NMR) analysis was performed for PIM-1 (in chloroform-*d*) and cPIM-1 (in DMSO-*d*_6_) by using a Bruker Avance II 500 MHz instrument.

The weight-average molar mass *M*_w_, number -average molar mass *M*_n_, and dispersity *Đ* of the PIM-1 sample were determined using multidetector gel permeation chromatography (GPC). PIM-1 was prepared as a 1 mg mL^–1^ chloroform solution. The solution was prefiltered using a polytetrafluoroethylene (PTFE) membrane filter (0.45 μm, Fisherbrand) and analyzed using a Viscotek VE2001 SEC solvent/sample module with two PL Mixed B columns (35 °C) and a Viscotek TDA 302 triple detector array (refractive index, light scattering, viscosity detectors). The system was calibrated using a 110 kg mol^–1^ polystyrene standard, and data were analyzed using OmniSEC software.

Elemental analysis of cPIM-1 polymers was performed using a Flash 2000 organic elemental analyzer (Thermo Scientific, The Netherlands), giving the elemental content of C, H, and N. The degree of hydrolysis was calculated by eq 1 based on the N/C ratio:^7^
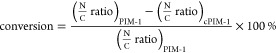
1

### 2.4 Membrane Preparation

Self-standing membranes were made from casting solutions prepared from 150 mg of polymer dissolved in 5 mL of THF under stirring. After filtration through glass wool, the solutions were poured into polytetrafluoroethylene (PTFE) Petri dishes and then covered over with a larger glass Petri dish to allow slow solvent evaporation for 3 days in a nitrogen atmosphere storage cabinet. The membranes were dried further in a 120 °C vacuum oven for 2 days.

Thin film composite (TFC) membranes were prepared using a kiss-coating method.^7^ The setup is shown in Figure S1a. PAN support was cut into a 4.5 cm × 10 cm rectangular sheet and then attached to the roller wheel with edges sealed with aluminum tape. The roller wheel was connected to a motor that was driven by a DC power supply (RS-3005P, RS PRO, UK) at a voltage of 13 V. PIM-1 and cPIM-1 solutions were prepared as 3% or 4% w/v in THF. During film coating, the contact between solution and support was controlled via surface tension, as shown in Figure S1b. After coating, the sheets were peeled off from the roller and stored in a nitrogen atmosphere storage cabinet at room temperature for around 18 h before testing.

### 2.5 Gas Permeation Tests

Gas permeance tests were performed at temperatures from 25 to 85 °C by the standard variable volume method.^38^ Single gas tests were performed in the sequence of N_2_, CH_4_, and CO_2_ with absolute feed pressure maintained at 40 psi and permeate side at atmospheric pressure. A permeation apparatus with an active area of 4.3 cm^2^ was located inside an oven. Data were collected after at least 10 min conditioning upon changing pressures and temperatures, and the time for a specified volume of gas to permeate through the membrane was recorded. Membrane permeance was calculated based on eq 2:
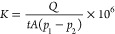
2*K* is the gas permeance (GPU, 1 GPU = 10^–6^ cm^3^ [STP] cm^–2^ s^–1^ cmHg^–1^ = 3.348 × 10^–10^ mol m^–2^ s^–1^ Pa^–1^), *t* is the permeation time (s), *Q* is the volume of gas that permeates through the membrane during the permeation time (cm^3^, corrected to STP [0 °C, 1 atm]), *A* is the active permeation area (cm^2^), and *p*_1_ and *p*_2_ are the pressure in the membrane feed side and permeate side (cmHg), respectively.

The membrane gas permeability was calculated by eq 3:
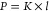
3where *P* is the permeability (barrer, 1 barrer = 10^–10^ cm^3^ [STP] cm cm^–2^ s^–1^ cmHg^–1^ = 3.348 × 10^–16^ mol m m^–2^ s^–1^ Pa^–1^) and *l* is the membrane thickness (μm).

The membrane selectivity was calculated as the ratio of gas permeances by eq 4:
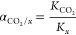
4where *x* is either N_2_ or CH_4_.

In Section 3.5, mixed gas permeation tests were performed using an equimolar feed mixture of CO_2_/N_2_ and CO_2_/CH_4_ at 40 and 60 psi for TFC membranes and at 40 and 80 psi for self-standing membranes (absolute pressure), with permeate side maintained at atmospheric pressure. Helium was used as a sweep gas for self-standing membranes, and no sweep gas was used for TFC membranes. The temperature was increased from 25 to 85 °C, and at each temperature, pressure was increased from low to high. Two different membranes were tested for each gas pair. The gas mixtures were analyzed using a 490 microGC (Agilent, USA) equipped with a PoraPLOT (PPU) column. Mixed gas permeance was calculated by eq 5:
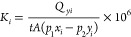
5where *i* represents either N_2_, CH_4_, or CO_2_; *x* and *y* are the mole fractions on the feed side and the permeate side, respectively.

In Section 3.6, TFC membranes were first conditioned under pure N_2_ feed for 50 min around 5 bar under different temperatures of 25, 50, and 75 °C, and then the mixed gas permeation experiment was performed using CO_2_/N_2_ feed (10%/90%) with total pressure gradually increased from 2.5 to 10 bar (absolute pressure); analysis and calculations are as mentioned above.

### 2.6 Scanning Electron Microscopy (SEM) Analysis

A scanning electron microscope (FEI Quanta 250 FEG-SEM) was used to characterize the cross section of PIM-1 and cPIM-1 TFC membranes before and after heat treatment at 85 °C for 2.5 h. Samples for SEM analysis were prepared by immersing them in DI water for 15 s and then introducing them into liquid nitrogen for another 15 s for sample fracture. The samples were coated with 5 nm of Au/Pd (80:20) nanoparticles using a Pt/Au Quorum Sputter (UK) and then left for drying for 3 h. The images were produced by utilizing a secondary electron (SE) detector. ImageJ software was used to measure the active layer thicknesses.

## 3 Results and Discussion

### 3.1 Polymer Characterization

The PIM-1 has mainly disubstituted structures, as there are neglectable shoulder peaks adjacent to aromatic proton peaks (a and b) in the ^1^H NMR spectrum, shown in Figure S2. From GPC, the *M*_w_, *M*_n_, and *Đ* values of PIM-1 are 96,000 g mol^–1^, 41,000 g mol^–1^, and 2.3, respectively. The elemental content of cPIM-1 is summarized in Table S1, with hydrolysis degree calculated based on eq 1. cPIM-1 polymers are named cPIM-1-*X*, where *X* represents the hydrolysis degree. cPIM-1-55% and cPIM-1-66% are used in experiments related to Section 3.6, while cPIM-1-68% is used in the other sections. As the hydrolysis degrees of all cPIM-1s are similar, the ^1^H NMR spectrum of cPIM-1-68% is presented in Figure S3 as an example. After acid hydrolysis, the broad carboxylic acid peak shown around 13–14 ppm proved successful chemical functionalization.

### 3.2 Single Gas Permeation

**Figure 2:**
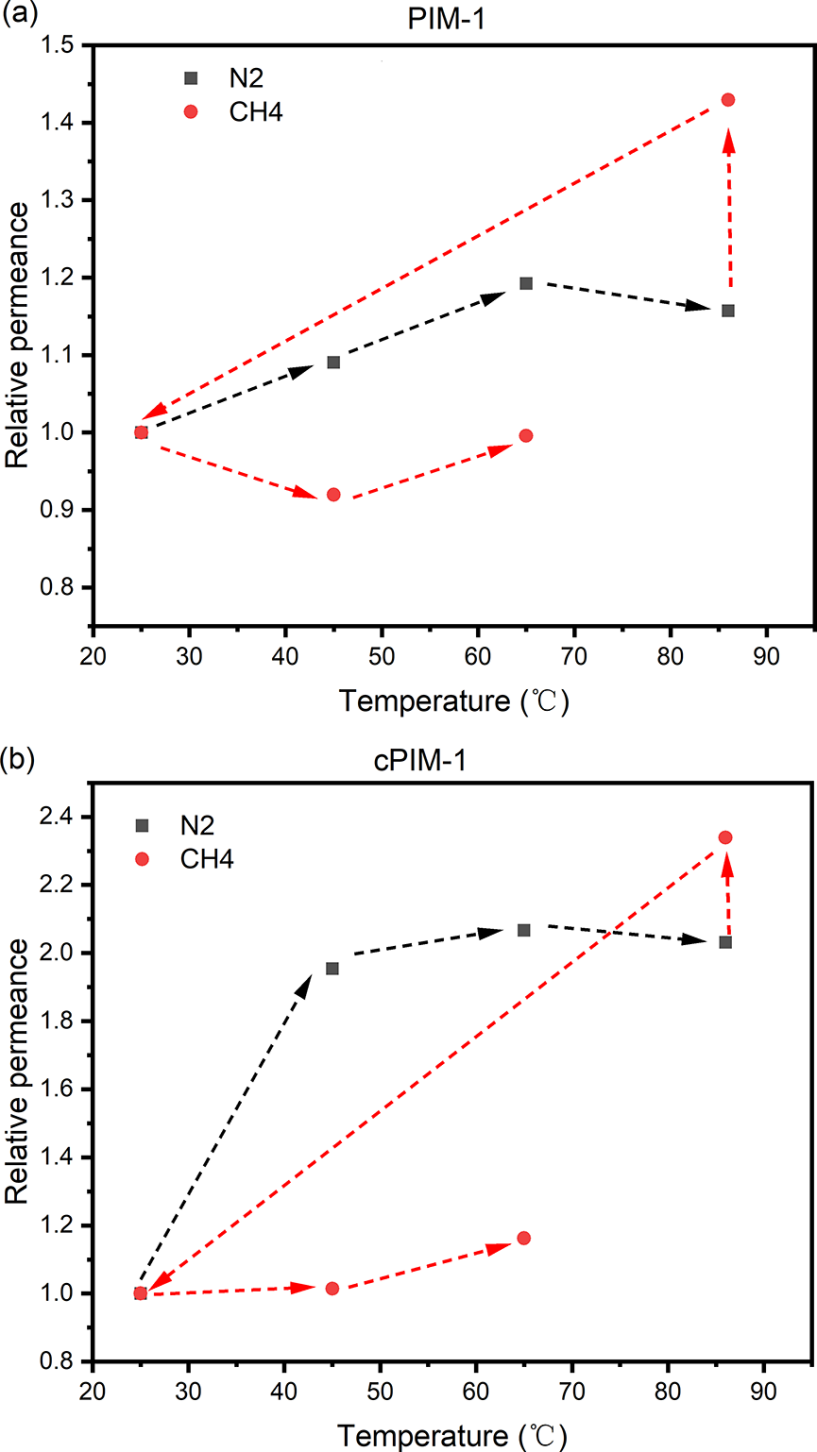
Change in N_2_ and CH_4_ permeance relative to that measured at 25 °C with the temperature for (a) PIM-1 and (b) cPIM-1 TFC membranes. The arrows indicate the testing sequence.

TFC membranes of PIM-1 and cPIM-1 were first tested under single gas conditions in the sequence of N_2_, CH_4_, and CO_2_. For each individual gas, the temperature was changed in a random order between 25 and 85 °C. A single membrane was tested across different single gas feeds and temperatures. The sequences of testing are provided in Figure 2 and Table S2 to help understand the effect of temperature better. Gas permeances exhibited some disparity with trends previously observed with self-standing PIM-1 films,^19^ where N_2_ and CH_4_ permeances were reported to be diffusion dominated and roughly proportional to the temperature change within the range from 25 to 55 °C.

As shown in Figure 2 and Table S2, starting with N_2_, the N_2_ permeance increased as the temperature increased from 25 to 65 °C. However, a slight decrease of N_2_ permeance was observed when the temperature was increased further from 65 to 85 °C. This could be an effect of accelerated physical aging that led to a decrease in permeance. The gas was then switched from N_2_ to CH_4_, while the temperature was maintained at 85 °C. A significant drop in CH_4_ permeance was observed when decreasing the temperature from 85 to 25 °C. Although a decrease in CH_4_ permeance with decreasing temperature is expected, the TFC membrane might still undergo physical aging as temperature decreased, which could significantly reduce the subsequent permeance tested at 25 °C. The change in CO_2_ permeance may be more complex, since in the study by Li et al.^19^ the CO_2_ permeability of PIM-1 self-standing membranes did not vary monotonically with temperature due to the combined effects of diffusivity and solubility coefficient change. In our experiments, TFC membranes could also be affected by accelerated physical aging.

A later experiment, in which the temperature of 85 °C was excluded, resulted in changes for which it was not necessary to account for excessive physical aging and, so, included proportional increases in permeance of N_2_ and CH_4_ with temperature. However, the aging effect on cPIM-1 was still notable, with lower CO_2_ permeance observed after around 1 h heat treatment history at 65 °C (∼400 GPU) compared with fresh membranes tested at room temperature (∼800 GPU) (Table S3 and Table S4).

### 3.3 Temperature Accelerated Physical Aging

**Figure 3:**
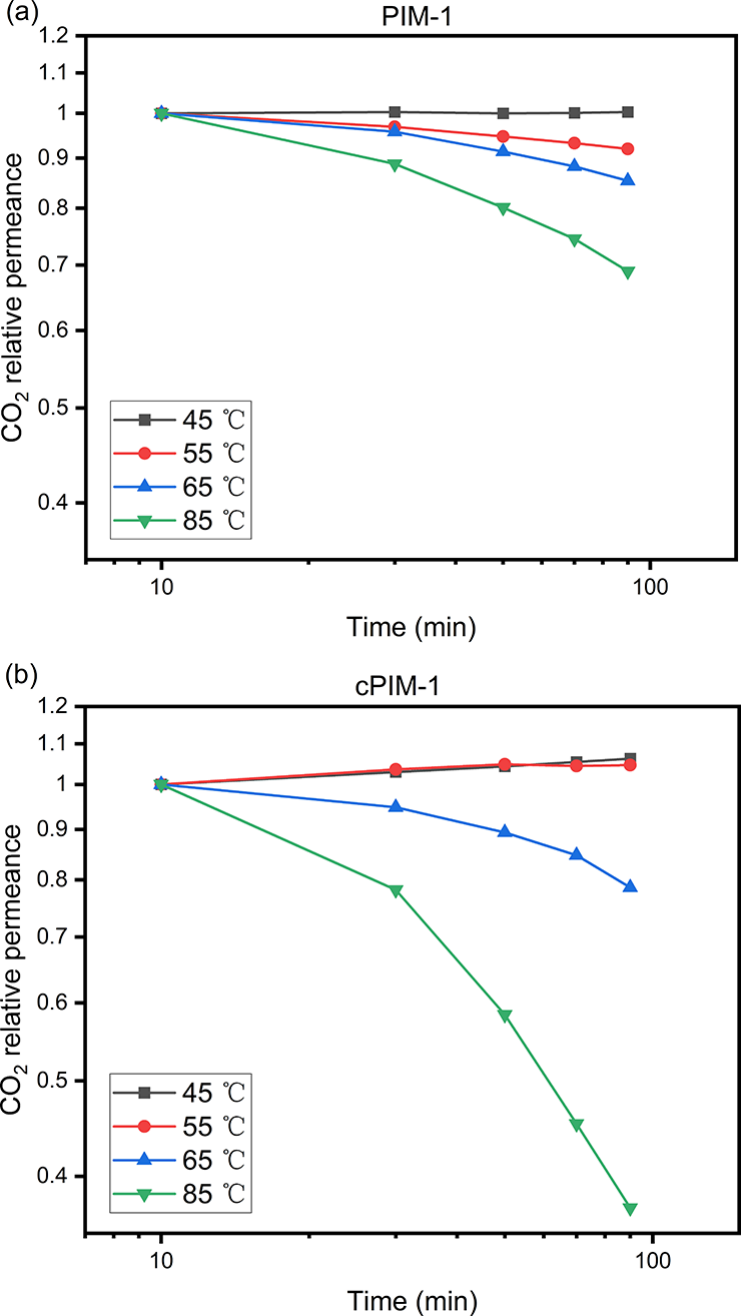
Dependence on time of the CO_2_ permeance relative to the permeance measured at 10 min, for temperatures from 45 to 85 °C, of TFC membranes of (a) PIM-1 and (b) cPIM-1.

To further study the effects of temperature-induced aging on thin film performance, the pure CO_2_ permeances of TFC membranes were monitored continuously for 90 min at different temperatures between 45 and 85 °C for individual membranes. As shown in Figure 3 and Table S4, physical aging accelerated at 55 °C for PIM-1, but the accelerated aging of cPIM-1 at 55 °C was offset by slight plasticization, which dominated CO_2_ permeation at 45 °C. The aging rates (β_p_) of thin films (−∂[log(*P*)]/∂[log(*t*)])^16^ were calculated using the last three time points. As presented in Table 1, the aging rates increased significantly with temperature and were much faster than that of normally aged thin films. cPIM-1 TFC membranes aged much more rapidly than PIM-1 at the same temperature. It should be noted that the minus values of β_p_ at 45 °C are because of testing error for both polymers and, in addition, a significant plasticization effect for cPIM-1. In this small time scale (1.5 h) of gas permeation testing at 45 °C, it is hard to see a significant permeance drop corresponding to normal physical aging, which explains the difference in β_p_ values between this work and literature under much longer aging intervals.^18,39^

**Table 1:** Comparison of Aging Rates for TFC Membranes of PIM-1, CPIM-1, and Trip (Me_2_)-TB at Different Temperatures

	aging rate (β_p_)			
*T* (°C)	PIM-1	cPIM-1	Trip (Me_2_)-TB	ref
45	–0.1	–0.5		
55	0.9	0.1		
65	2.0	3.8		this work^b^
85	4.4	13.2		
85^a^	5.9	11.2		
25	0.7			(39)
25			1.0	(18)
90			15.7	(18)

a Aging rates of TFC membranes under direct heat treatment in an oven at 85 °C for 2.5 h in air (Figure 6) were calculated for comparison to TFC membranes heat treated in a membrane cell under continuous testing.

b TFC membranes were stored at room temperature around 18 h before thermal treatment studies commenced.

### 3.4 Mixed Gas Permeation

**Figure 4:**
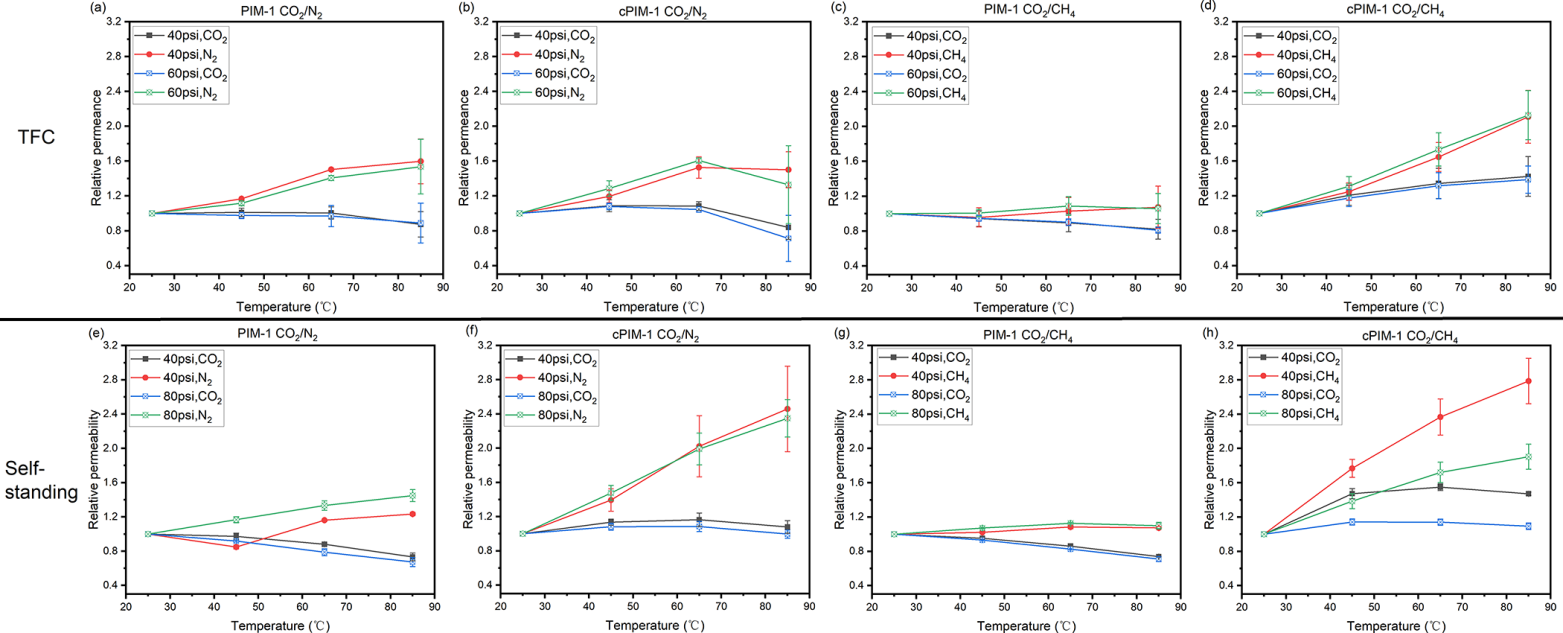
CO_2_/N_2_ mixed gas separation performance of TFC membranes of (a) PIM-1 and (b) cPIM-1, self-standing membranes of (e) PIM-1 and (f) cPIM-1, CO_2_/CH_4_ mixed gas separation performance of TFC membranes of (c) PIM-1 and (d) cPIM-1, and self-standing membranes of (g) PIM-1 and (h) cPIM-1. All pressures indicate absolute pressure at the feed side with the permeating side maintained at atmospheric pressure.

Both TFC and self-standing thick membranes of PIM-1 and cPIM-1 were further tested with equimolar gas mixtures of CO_2_/N_2_ and CO_2_/CH_4_ in a temperature range from 25 to 85 °C, with the overall performance presented in Figure 4 and Tables S5–S8. It should be noted that all self-standing membranes had a post-treatment process of drying in a vacuum oven at 120 °C for 2 days to remove the residual solvent. The performance of TFC membranes correlated well with that of the self-standing membranes. The effect of pressure was much less significant than temperature. All membranes exhibited significantly decreased mixed gas selectivities at elevated temperatures (Tables S5–S8), which is typically a consequence of an increase in the diffusion coefficient and a decrease in the solubility coefficient. For the CO_2_/N_2_ mixture in PIM-1 TFC membranes, the transport of N_2_ was dominated by diffusion, and the permeance increased as expected when temperature increased, while for the permeation of CO_2_, the increase in diffusion coefficient was compensated by the decrease in solubility coefficient, so CO_2_ permeance exhibited little relative change across the experiment, which aligned with a previous report.^19^ In this case, the decrease in CO_2_/N_2_ permselectivity as temperature increases was largely the result of the significantly decreased diffusivity selectivity and relatively stable solubility selectivity (Table 2). cPIM-1 has similar solubility selectivity and slightly higher diffusivity selectivity (due to a better size sieving effect) than PIM-1;^33^ thus, similar temperature-dependent mixed gas permeation behaviors were observed, as expected. Since the residence time at each temperature is short (approximately 0.5 h), the slight accelerated aging was only observable at 85 °C. However, in the case of CO_2_/CH_4_, compared with N_2_, the permeation of CH_4_ is more affected by the solubility term, resulting in a stronger competitive sorption with decreased permeances of both components in PIM-1 TFC membranes (Figure 4c). For cPIM-1, the existence of two sorbing penetrants leads to significant plasticization at higher temperatures, contributing to an increase in diffusion coefficients and bringing about a more than 100% increase in CH_4_ permeance and a slight increase in CO_2_ permeance. In this case, the extra decrease in diffusivity selectivity induced by plasticization^40^ also contributes to the bad separation performance.

**Table 2:** Temperature-Dependent Pure Gas Diffusion (*D*) and Sorption (*S*) Coefficients and Selectivities Measured at 2 Atm of PIM-1 and CPIM-1 Reported in the Literature

		*D* (10^–6^ cm^2^ s^–1^)			*S* (cm^3^_SPT_ cm^–3^_pol_ atm^–1^)			CO_2_/N_2_		CO_2_/CH_4_		
polymer	*T* (°C)	N_2_	CH_4_	CO_2_	N_2_	CH_4_	CO_2_	α_D_^pure^	*S*_D_^pure^	α_D_^pure^	*S*_D_^pure^	ref^a^
PIM-1	25	0.74	0.29	1.45	2.56	8.17	26.82	1.96	10.49	4.94	3.28	(19)
	35	1.03	0.46	1.93	2.20	6.66	21.70	1.88	9.86	4.18	3.26	
	45	1.40	0.71	2.25	1.86	5.59	18.61	1.61	9.99	3.16	3.33	
	55	1.83	0.96	2.73	1.62	4.72	14.89	1.49	9.22	2.84	3.15	
PIM-1	35	1	0.38	1.15	3	12.15	48.5	1.15	16.17	3.03	4	(33)
cPIM-1	35	0.17	0.048	0.241	2.55	10.2	44	1.42	17.25	5.02	4.32	
PIM-1	–30	0.01	0.002	0.04	19.3	81.3	278.9	5.77	14.45	19.74	3.43	(20)
	–20	0.02	0.01	0.07	17.4	57.5	206.7	4.71	11.88	13.36	3.60	
	–10	0.03	0.01	0.12	13.3	50.2	155.8	3.58	11.71	11.71	3.11	
	0	0.06	0.02	0.17	9.5	32.5	120.8	2.83	12.72	7.54	3.72	
	10	0.11	0.04	0.25	7.3	26.4	95.8	2.24	13.04	6.11	3.62	
	30	0.26	0.11	0.47	5.4	18.4	61.4	1.84	11.46	4.48	3.34	

a Data from ref (33) are taken from the table as reported, while data from refs (19) and (20) are taken from figures and recalculated based on the solution-diffusion model (*P* = *SD*).

### 3.5 Plasticization of TFC Membranes

**Figure 5:**
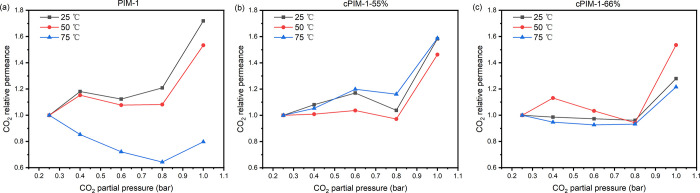
Effect of the CO_2_ partial pressure (0.25–1 bar) on the CO_2_ permeance relative to that measured at 0.25 bar, at different temperatures, for (a) PIM-1, (b) cPIM-1-55%, and (c) cPIM-1-66% TFC membranes.

It was reported previously that PIM-1 and cPIM-1 TFC membranes exhibited instant CO_2_ permeance increase with increasing pressure from 1.5 bar, which indicated a significant plasticization effect.^7,41^ To better study the plasticization behavior of PIM-1 and cPIM-1 TFC membranes, a mixed gas permeation study was performed using gas mixtures with a lower concentration of CO_2_ (10%/90% CO_2_/N_2_) to provide insights into the gas permeation behavior in a lower CO_2_ partial pressure range. All membranes (PIM-1, cPIM-1-55%, and cPIM-1-66%) were conditioned first at different temperatures (25, 50, 75 °C) for 50 min and then tested under mixed gas feed, with absolute pressure increasing from 2.5 to 10 bar. As shown in Figure 5 and Table S9, all TFC membranes exhibited a significant permeance jump when CO_2_ partial pressure increased from 0.8 to 1 bar, which indicated the beginning of plasticization, with plasticization pressure around 1 bar. The plasticization pressure is independent of the testing temperature and hydrolysis degree of PIM-1.

In Figure 5a it can be seen that at 75 °C, PIM-1 shows a permeance drop as the CO_2_ partial pressure increases up to 0.8 bar. This may be attributed to temperature-induced physical aging. Though all membranes are conditioned before mixed gas testing, it might not be sufficient to significantly densify PIM-1 TFC membranes, as they have slower accelerated aging rate compared with cPIM-1 TFC membranes. Nevertheless, plasticization overcomes physical aging at a CO_2_ partial pressure above 0.8 bar.

Mixed gas selectivity exhibited a significant drop when temperature shifted from 25 to 50 °C, which aligns with the results in Section 3.3. The selectivity changes between 50 and 75 °C are relatively small, which may be because the accelerated physical aging, which raises selectivity, offsets the decline in selectivity caused by the temperature increase.

### 3.6 Methanol Vapor Treatment

**Figure 6:**
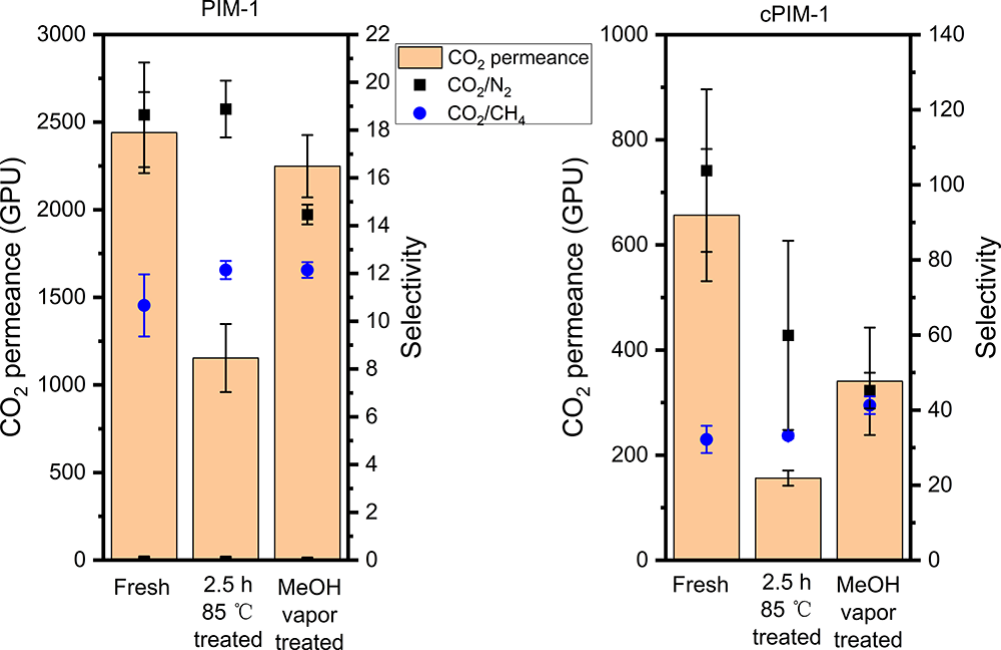
Single gas separation performance (CO_2_ permeance, CO_2_/N_2_ and CO_2_/CH_4_ selectivity) of PIM-1 and cPIM-1 TFC membranes tested fresh, after 2.5 h in an 85 °C oven, and after 5 days of methanol vapor rejuvenation.

**Figure 7:**
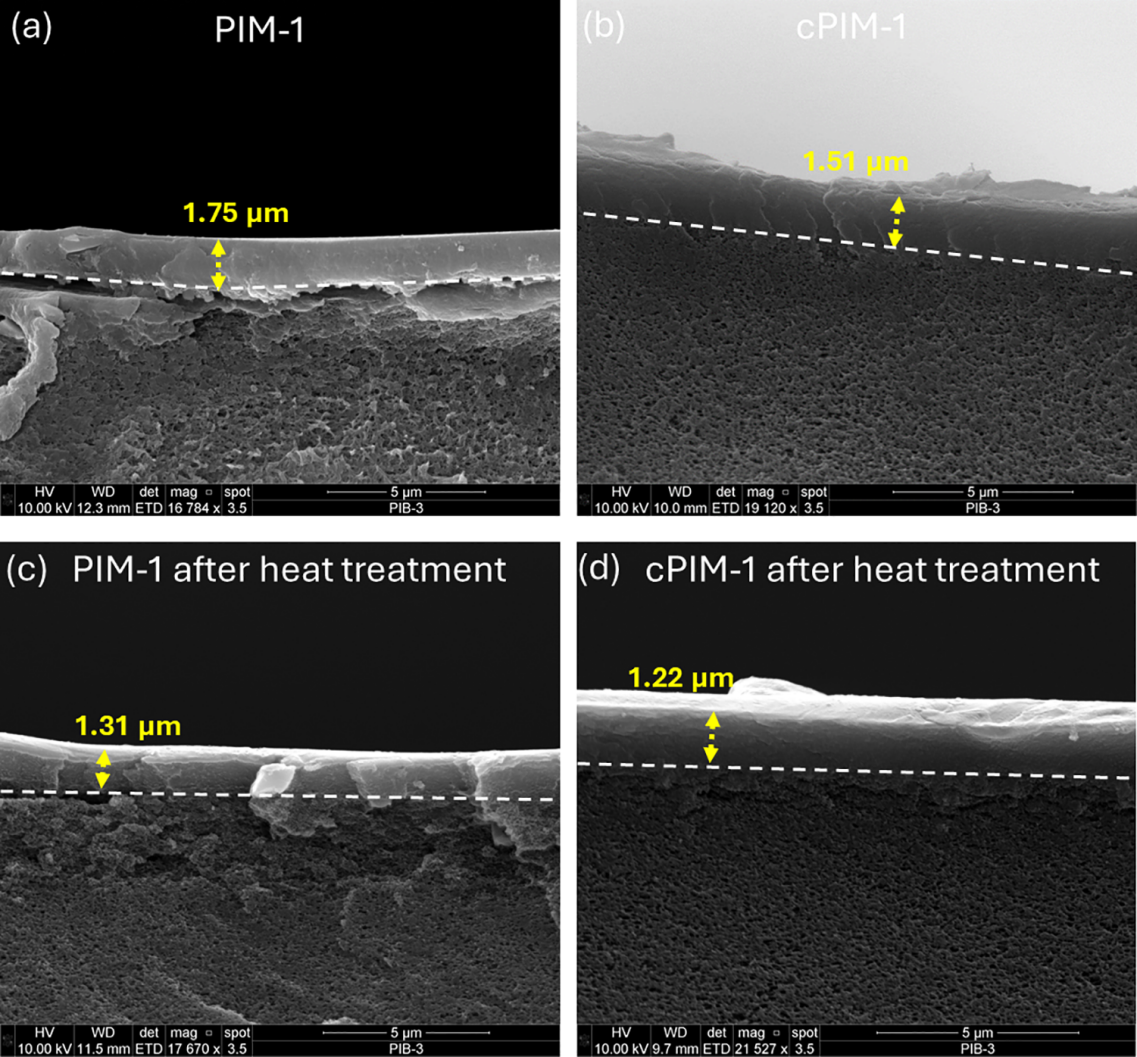
Cross-sectional SEM images of (a) fresh PIM-1 and (b) fresh cPIM-1 TFC membranes and (c) PIM-1 and (d) cPIM-1 TFC membranes after heat treatment at 85 °C for 2.5 h. The active layer thickness is highlighted in yellow, with white dashed lines marking the boundaries between the active layers and the support layers.

Finally, a methanol vapor treatment^42^ was performed to determine whether the physical aging induced at higher temperatures was reversible. To ensure that the testing history did not affect the recovery efficacy, fresh TFC membranes were directly placed in an 85 °C oven for 2.5 h. As presented in Figure 6 and Table S10, a reduction in permeance was observed after heat treatment, similar to that presented in Figure 3 and Table 1. After 5 days of methanol vapor rejuvenation, the performance of PIM-1 TFC membranes was nearly restored to that of the fresh membranes, indicating that there was no effect other than physical aging induced by temperature in the short term. However, for cPIM-1 TFC membranes, rejuvenation was not as effective as with PIM-1, and only half of the initial CO_2_ permeance was recovered. It is possible that the polymer densification of the cPIM-1 structure was more significant than that in PIM-1 (76% vs 53% CO_2_ permeance reduction). A key difference between PIM-1 and cPIM-1 is the potential for intra- and intermolecular hydrogen bonding in cPIM-1. At elevated temperatures, we can expect disruption of the hydrogen-bonded structure of cPIM-1, giving rise to an additional driving force for densification. Such a gentle methanol vapor treatment was insufficient to fully refresh them. Cross-sectional SEM images of the PIM-1 and cPIM-1 TFC membranes before and after thermal treatment are presented in Figure 7. All TFC membranes exhibit active layer thicknesses ranging from 1 to 2 μm. Notably, the active layer thickness decreases following heat treatment, indicating densification of the polymeric structure.

## 4 Conclusions

In conclusion, we investigated the effect of temperature on the TFC membranes of PIM-1 and cPIM-1. Physical aging first became significant at 55 °C and tended to accelerate at higher temperatures. cPIM-1, which was shown to have a strong plasticization tendency in a previous study, was more sensitive to the temperature than PIM-1. However, this effect could be at least partially mitigated by methanol vapor refreshment. In mixed gas tests, the separation efficacy typically deteriorated as temperatures rose. In prior work it was noted that the decreased permselectivities at elevated temperatures^19^ and increased permselectivities at subambient temperatures^20^ were primarily influenced by the more significant changes in diffusivity selectivity rather than solubility selectivity. All TFC membranes developed in this work exhibited a CO_2_ plasticization pressure around 1 bar, regardless of the environmental temperature and degree of hydrolysis. Further work needs to focus on examining the performance of membranes under industrial gas separation conditions and improving the aging and plasticization resistance of PIM-1-based TFC membranes.

## Data Availability

Data supporting this study are available within the article and the Supporting Information.

## Supporting Information Available

Kiss-coating set-up, ^1^H NMR spectra, elemental analysis of cPIM-1 and gas permeation data (PDF)
