# Supplementary material for: Effect of Temperature-Induced Aging on the Gas Permeation Behavior of Thin Film Composite Membranes of PIM-1 and Carboxylated PIM-1
Source: Ind Eng Chem Res. 2024 Sep 4;63(37):16198–207. doi: 10.1021/acs.iecr.4c02230 (PMC11417989; doi:10.1021/acs.iecr.4c02230)
Supplement: Supplementary file 1 — ie4c02230_si_001.pdf [file ie4c02230_si_001.pdf]

Supporting information

Effect of temperature-induced aging on the gas permeation behavior of thin film composite membranes of PIM-1 and carboxylated PIM-1

Ming Yu [1,2], Andrew B. Foster [2], Mustafa Alshurafa [2], Colin A. Scholes [1]*, Sandra E. Kentish [1], and Peter M. Budd [2]*

1 Department of Chemical Engineering, The University of Melbourne, Melbourne, VIC. 3010, Australia.

2 Department of Chemistry, School of Natural Sciences, The University of Manchester, M13 9PL Manchester, U.K.

**Corresponding authors:* Peter.Budd@manchester.ac.uk, cascho@unimelb.edu.au

Contents

Figure S1. (a) Kiss-coating setup composed of a steel coater connected to a motor, a steel bucket for containing coating solution, and glass plates for adjusting the distance between PAN support and solution, and (b) the contact between solution and support was via surface tension. 3

Figure S2. ^{1}H NMR spectrum of PIM-1 3

Figure S3. ^{1}H NMR spectrum of cPIM-1-68% 4

Table S1. Elemental content of cPIM-1 polymers, polymers are named as cPIM-1-X, where X is the hydrolysis degree. 4

Table S2. Temperature dependent single gas permeance of PIM-1 and cPIM-1 TFC membranes following the order of N_2, CH_4 and CO_2, Temperature was changed randomly within the range from 25 to 85 °C, in the sequence given. 4

Table S3. Temperature dependent single gas permeance of PIM-1 and cPIM-1 TFC membranes following the order of N_2, CH_4 and CO_2, Temperature was changed randomly within the range from 25 to 65 °C, in the sequence given. 5

Table S4. Pure CO_2 permeance of PIM-1 and cPIM-1 TFC membranes at different temperatures........ 5

Table S5. CO_2/N_2 mixed gas separation performance of PIM-1 and cPIM-1 TFC membranes at 40 psi and 60 psi, (equimolar feed, data are averaged for two coupons, with standard deviation). 6

Table S6. CO_2/N_2 mixed gas separation performance of PIM-1 and cPIM-1 self-standing membranes at 40 psi and 80 psi, (equimolar feed, data are averaged for two coupons, with standard deviation). 6

Table S7. CO_2/CH_4 mixed gas separation performance of PIM-1 and cPIM-1 TFC membranes at 40 psi and 60 psi, (equimolar feed, data are averaged for two coupons, with standard deviation). 7

Table S8. CO_2/CH_4 mixed gas separation performance of PIM-1 and cPIM-1 TFC self-standing membranes at 40 and 80 psi, (equimolar feed, data are averaged for two coupons, with standard deviation). 7

Table S9. Mixed gas separation performance of PIM-1, cPIM-1-55% and cPIM-1-66% TFC membranes using 10%/90% CO_2/N_2 feed at different temperatures with absolute feed pressure increased from 2.5 bar to 10 bar. 8

Table S10. Ideal gas separation performance of TFC membranes of PIM-1 and cPIM-1 at different stages in their treatment history (fresh, 2.5 h heat treatment in an 85°C oven and followed with a 5-day methanol vapor treatment). 8

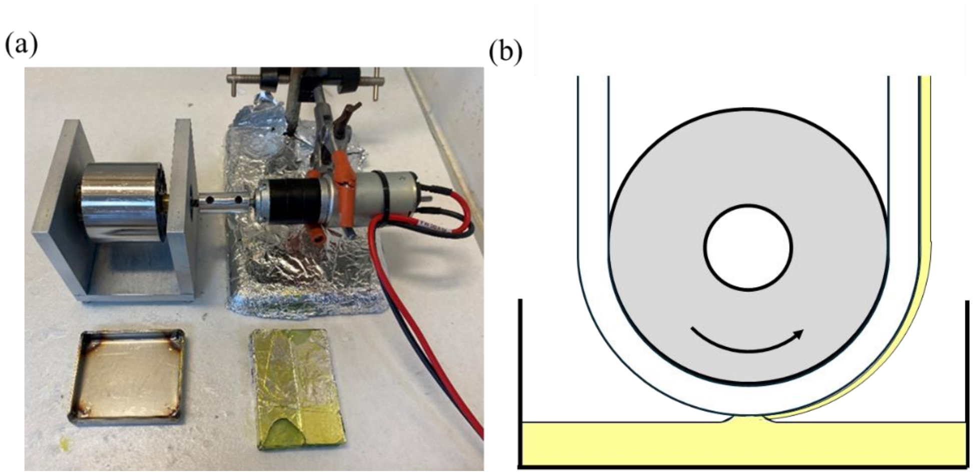

Figure S1. (a) Kiss-coating setup composed of a steel coater connected to a motor, a steel bucket for containing coating solution, and glass plates for adjusting the distance between PAN support and solution, and (b) the contact between solution and support was via surface tension.

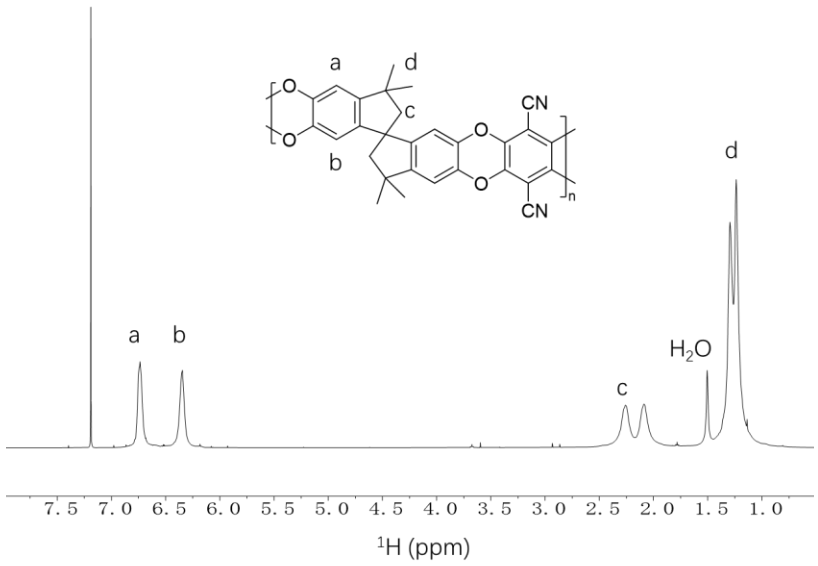

Figure S2. ^{1}H NMR spectrum of PIM-1

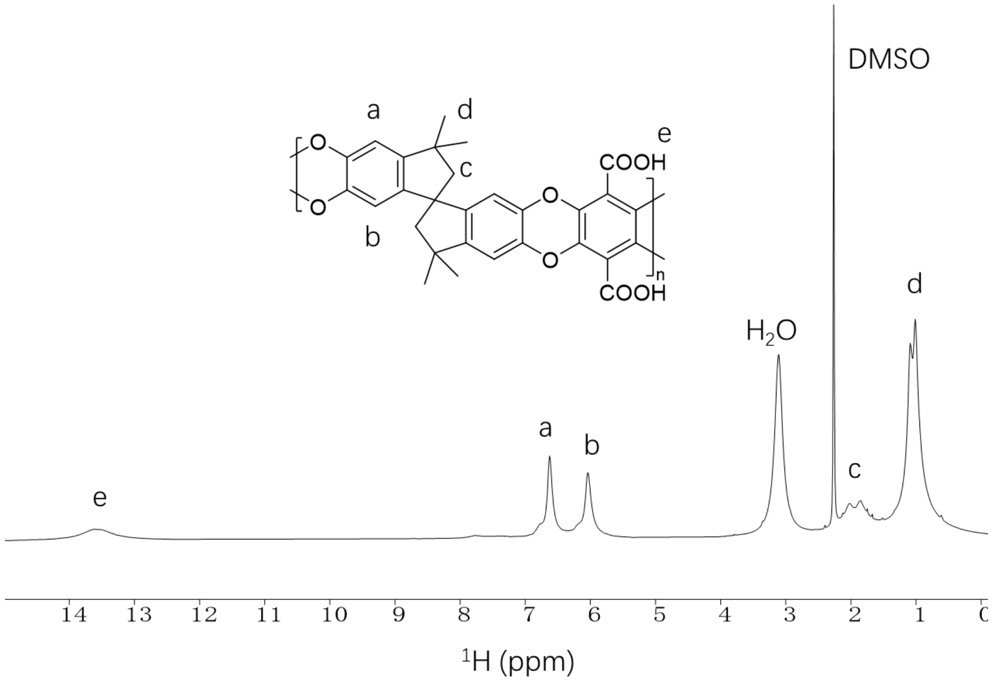

Figure S3. ^{1}H NMR spectrum of cPIM-1-68%

Table S1. Elemental content of cPIM-1 polymers, polymers are named as cPIM-1-X, where X is the hydrolysis degree.

Polymer	Reaction time (h)	C	H	N	hydrolysis degree
cPIM-1-55%	12	68.92%	4.49%	2.51%	55%
cPIM-1-66%	24	67.09%	4.62%	1.84%	66%
cPIM-1-68%	24	64.21%	4.21%	1.66%	68%

Table S2. Temperature dependent single gas permeance of PIM-1 and cPIM-1 TFC membranes following the order of N_2, CH_4 and CO_2, Temperature was changed randomly within the range from 25 to 85 °C, in the sequence given.

	T	N_2	sequence	CH_4	sequence	CO_2	sequence
	℃	GPU		GPU		GPU	
PIM-1 TFC	25	101	1	134	2	1344	4
	45	110	2	123	3	1300	3
	65	120	3	134	4	1039	1
	85	117	4	192	1	1222	2
cPIM-1 TFC	25	15	1	15	2	287	4
	45	30	2	15	3	301	3
	65	31	3	18	4	312	1
	85	31	4	36	1	327	2

Table S3. Temperature dependent single gas permeance of PIM-1 and cPIM-1 TFC membranes following the order of N_2, CH_4 and CO_2, Temperature was changed randomly within the range from 25 to 65 °C, in the sequence given.

	T	N_2	sequence	CH_4	sequence	CO_2	sequence
	°C	GPU		GPU		GPU	
PIM-1 TFC	25	107	1	220	3	1845	1
	45	118	2	243	2	1820	2
	65	131	3	258	1	1787	3
cPIM-1 TFC	25	16	1	26	3	404	1
	45	18	2	32	2	415	2
	65	24	3	38	1	456	3

Table S4. Pure CO_2 permeance of PIM-1 and cPIM-1 TFC membranes at different temperatures.

		CO_2 permeance (GPU)			
		Temperature (°C)			
	Time (min)	45	55	65	85
PIM-1 TFC	10	1569	1547	1732	1421
	30	1573	1499	1659	1261
	50	1569	1465	1583	1138
	70	1570	1442	1529	1057
	90	1573	1423	1478	980
cPIM-1 TFC	10	899	777	785	811
	30	926	805	744	634
	50	938	815	701	474
	70	948	812	665	367
	90	955	813	617	301

Table S5. CO_2/N_2 mixed gas separation performance of PIM-1 and cPIM-1 TFC membranes at 40 psi and 60 psi, (equimolar feed, data are averaged for two coupons, with standard deviation).

		40 psi			60 psi		
	Temper-ature	CO_2	N_2	CO_2/N_2	CO_2	N_2	CO_2/N_2
	℃	GPU	GPU		GPU	GPU	
PIM-1 TFC	25	620±100	25±0	25±3	570±20	30±2	20±5
	45	620±70	29±0	22±2	560±95	32±2	18±4
	65	615±55	38±0	17±1	550±45	40±4	14±2
	85	530±4	40±6	14±2	490±25	44±15	12±2
cPIM-1 TFC	25	340±85	13±6	27±5	330±75	14±6	26±6
	45	370±65	16±6	25±5	360±65	18±7	22±5
	65	370±75	20±7	19±3	350±85	22±10	17±3
	85	290±115	21±12	15±3	250±140	20±15	14±3

Table S6. CO_2/N_2 mixed gas separation performance of PIM-1 and cPIM-1 self-standing membranes at 40 psi and 80 psi, (equimolar feed, data are averaged for two coupons, with standard deviation).

		40 psi			80 psi		
	Temper-ature	CO_2	N_2	CO_2/N_2	CO_2	N_2	CO_2/N_2
	℃	barrer	barrer		barrer	barrer	
PIM-1 self-standing	25	5890	510	12	5800±30	360±1	16±0
	45	5730±230	440±12	13±0	5490±90	420±14	13±0
	65	5050±250	510±15	10±0	4710±270	480±20	10±0
	85	4200±430	540±20	8±0	4030±340	530±27	8±0
cPIM-1 self-standing	25	1810±390	60±5	30±9	1750±290	60±4	30±5
	45	2060±510	80±1	30±6	1919±490	90±10	20±3
	65	2120±590	120±10	20±3	1910±520	120±20	15±2
	85	1970±550	140±17	14±2	1760±470	150±20	12±1

Table S7. CO_2/CH_4 mixed gas separation performance of PIM-1 and cPIM-1 TFC membranes at 40 psi and 60 psi, (equimolar feed, data are averaged for two coupons, with standard deviation).

		40 psi			60 psi		
	Temper-ature	CO_2	CH_4	CO_2/CH_4	CO_2	CH_4	CO_2/CH_4
	°C	GPU	GPU		GPU	GPU	
PIM-1 TFC	25	1450±230	280±60	6±2	1270±370	280±50	5±3
	45	1380±350	280±30	6±2	1210±290	280±40	5±3
	65	1310±360	290±20	5±2	1160±380	300±30	4±2
	85	1260±360	300±1	4±1	1040±340	290±8	4±2
cPIM-1 TFC	25	160±60	20±6	9±1	160±60	20±6	8±1
	45	190±60	25±5	8±0	190±50	30±6	7±0
	65	210±50	30±6	7±0	210±50	35±7	6±0
	85	230±50	40±6	6±0	220±40	40±7	5±0

Table S8. CO_2/CH_4 mixed gas separation performance of PIM-1 and cPIM-1 TFC self-standing membranes at 40 and 80 psi, (equimolar feed, data are averaged for two coupons, with standard deviation).

		40 psi			80 psi		
	Temper-ature	CO_2	CH_4	CO_2/CH_4	CO_2	CH_4	CO_2/CH_4
	°C	barrer	barrer		barrer	barrer	
PIM-1 self-standing	25	4840±280	810±90	6±1	5040±640	780±190	7±1
	45	4610±320	830±90	6±1	4700±610	830±200	6±1
	65	4160±230	880±110	5±1	4160±530	880±210	5±1
	85	3570±230	870±110	4±1	3570±310	850±170	4±1
cPIM-1 self-standing	25	1600±130	110±20	14±4	2120±80	170±50	13±3
	45	2360±290	200±50	12±5	2430±160	240±80	11±3
	65	2480±270	270±80	10±4	2420±163	300±110	9±2
	85	2360±220	320±100	8±3	2320±160	330±120	7±2

Table S9. Mixed gas separation performance of PIM-1, cPIM-1-55% and cPIM-1-66% TFC membranes using 10%/90% CO_2/N_2 feed at different temperatures with absolute feed pressure increased from 2.5 bar to 10 bar.

Polymer	Pressure (bar)	25 °C Permeance (GPU) N_2	25 °C Permeance (GPU) CO_2	25 °C Selectivity	50 °C Permeance (GPU) N_2	50 °C Permeance (GPU) CO_2	50 °C Selectivity	75 °C Permeance (GPU) N_2	75 °C Permeance (GPU) CO_2	75 °C Selectivity
PIM-1 TFC membrane	2.5	31	400	13	47	475	10	24	230	10
	4	37	465	13	46	545	12	18	195	11
	6	39	445	11	45	510	11	16	165	11
	8	45	475	11	48	510	11	14	145	10
	10	66	675	10	64	725	11	17	180	11
cPIM-1-55% TFC membrane	2.5	37	550	15	46	470	10	9	110	12
	4	35	595	17	43	470	11	9	115	13
	6	35	645	18	42	485	12	10	130	13
	8	35	570	16	44	455	10	10	125	12
	10	48	870	18	58	685	12	13	170	13
cPIM-1-66% TFC membrane	2.5	10	230	24	28	320	12	25	245	10
	4	10	225	23	26	360	14	23	230	10
	6	9	225	25	25	330	13	22	225	10
	8	9	220	25	26	300	11	22	230	10
	10	12	295	25	37	490	13	28	300	10

Table S10. Ideal gas separation performance of TFC membranes of PIM-1 and cPIM-1 at different stages in their treatment history (fresh, 2.5 h heat treatment in an 85°C oven and followed with a 5-day methanol vapor treatment).

	Treatment	N_2	CH_4 (GPU)	CO_2	CO_2/N_2	CO_2/CH_4
PIM-1 TFC	fresh	130±30	230±50	2440±230	10±2	11±1
	2.5 h, 85 °C	60±6	100±13	1150±190	19±1	12±0
	5-day MeOH vapor	160±17	320±40	2250±180	14±0	7±0
cPIM-1 TFC	fresh	6±0	20±2	660±130	100±20	30±4
	2.5 h, 85 °C	3±1	5±1	160±14	60±25	33±1
	5-day MeOH vapor	8±3	8±3	340±100	45±5	41±2
